# DNA aptamers inhibit SARS-CoV-2 spike-protein binding to hACE2 by an RBD- independent or dependent approach

**DOI:** 10.7150/thno.74428

**Published:** 2022-07-18

**Authors:** Achut Prasad Silwal, Siddhartha Kalpa Samadhi Thennakoon, Satya Prakash Arya, Rick Mason Postema, Raunak Jahan, Chien Minh Tran Phuoc, Xiaohong Tan

**Affiliations:** Department of Chemistry and Center for Photochemical Sciences, Bowling Green State University, Bowling Green, Ohio 43403, United States.

**Keywords:** SARS-CoV-2, DNA aptamer, S2-protein, fusion aptamer, spike-protein

## Abstract

**Objective:** Nobody knows when the COVID-19 pandemic will end or when and where the next coronavirus will outbreak. Therefore, it is still necessary to develop SARS-CoV-2 inhibitors for different variants or even the new coronavirus. Since SARS-CoV-2 uses its surface spike-protein to recognize hACE2, mediating its entry into cells, ligands that can specifically recognize the spike-protein have the potential to prevent infection.

**Methods:** We have recently discovered DNA aptamers against the S2-domain of the WT spike-protein by exploiting the selection process called SELEX. After optimization, among all candidates, the aptamer S2A2C1 has the shortest sequence and the best binding affinity toward the S2-protein. More importantly, the S2A2C1 aptamer does not bind to the RBD of the spike-protein, but it efficiently blocks the spike-protein/hACE2 interaction, suggesting an RBD-independent inhibition approach. To further improve its performance, we conjugated the S2A2C1 aptamer with a reported anti-RBD aptamer, S1B6C3, using various linkers and constructed hetero-bivalent fusion aptamers. Binding affinities of mono and fusion aptamers against the spike-proteins were measured. The inhibition efficacies of mono and fusion aptamers to prevent the hACE2/spike-protein interaction were determined using ELISA.

**Results:** Anti-spike-protein aptamers, including S2A2C1 and S1B6C3-A5-S2A2C1, maintained high binding affinity toward the WT, Delta, and Omicron spike-proteins and high inhibition efficacies to prevent them from binding to hACE2, rendering them well-suited as diagnostic and therapeutic molecular tools to target SARS-CoV-2 and its variants.

**Conclusions:** Overall, we discovered the anti-S2 aptamer, S2A2C1, which inhibits the hACE2/spike-protein interaction via an RBD-independent approach. The anti-S2 and anti-RBD aptamers were conjugated to obtain the fusion aptamer, S1B6C3-A5-S2A2C1, which recognizes the spike-protein by an RBD-dependent approach. Our strategies, which discovered aptamer inhibitors targeting the highly conserved S2-protein, as well as the design of fusion aptamers, can be used to target new coronaviruses as they emerge.

## Introduction

**Figure 1:**
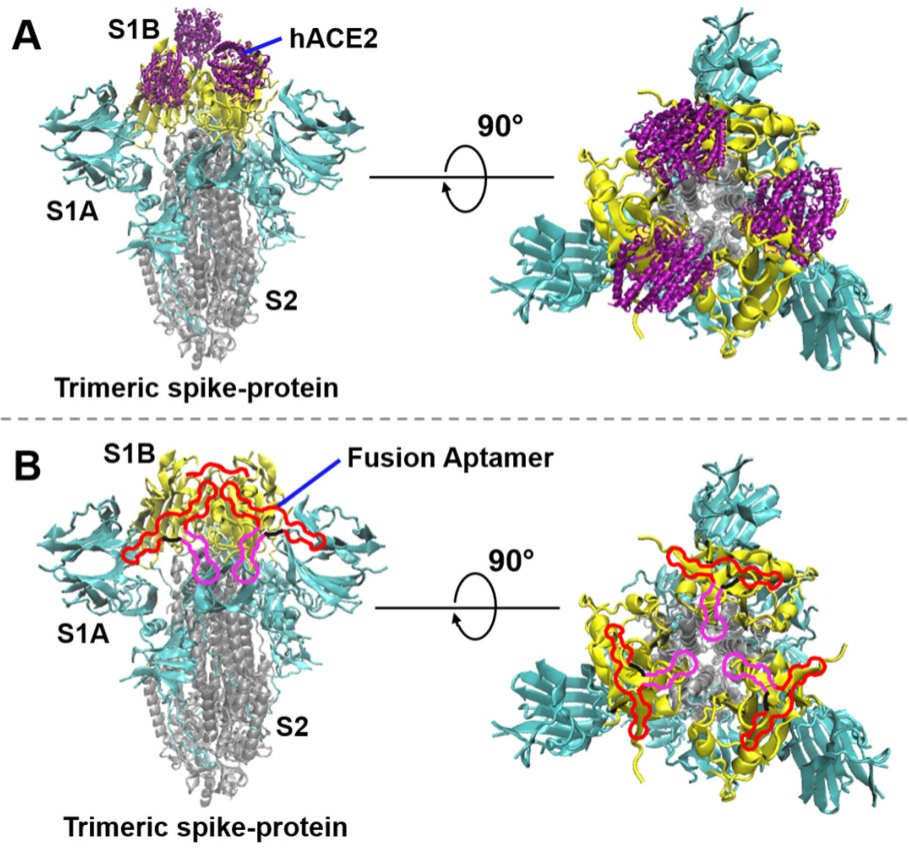
** The interaction of the SARS-CoV-2 spike-protein to hACE2 or fusion aptamers (not in scale). (A)** The interaction of the trimeric spike-protein and hACE2. The three S1B-domains, S1A-domains, and S2-subunits of the trimeric spike-protein are depicted in yellow, cyan, and silver colors, respectively (PDB code 6vxx). hACE2 (PBD code 6mOj) is depicted in purple color. **(B)** Demonstration of SARS-CoV-2 neutralization by using our fusion aptamers. In a single fusion aptamer, the S1B6C3 aptamer, linker, and S2A2C1 aptamer are depicted in red, black, and pink colors, respectively.

The Coronavirus Disease 2019 (COVID-19) pandemic, caused by severe acute respiratory syndrome coronavirus 2 (SARS-CoV-2), has presented one of the most dangerous global health care challenges in modern history. SARS-CoV-2 uses its homotrimer spike-protein (spike-protein) to attach to the host cell via human angiotensin converting enzyme 2 (hACE2). In humans, this attachment subsequently results in leukocytic infiltration, increased blood vessel permeability, alveolar wall permeability, and decreased secretion of lung surfactants. These adverse effects cause many respiratory problems. Moreover, the exacerbation of local inflammation causes a cytokine storm, eventually leading to a systemic inflammatory response syndrome 1-3. As a strategy to stop the viral infection, the interaction between the spike-protein and hACE2 can be blocked. Each spike-protein has S1 and S2 subunits; S1 contains two primary domains S1A and S1B (Figure 1). S1A determines the range of the host from the viral particle, and S1B, also known as the receptor-binding domain (RBD), establishes the direct interaction with hACE2 (Figure 1A) 4. On the other hand, the S2 subunit mediates the fusion of the viral membrane to its potential host cell via the heptad repeat regions. Studies have shown that virus entry is accomplished via a cascade of events: the S1-protein binds to hACE2, then the S2-protein undergoes a conformation change from a metastable pre-fusion to a more stable post-fusion state, allowing viral entry to the host cell 5-7. Due to the fact that RBD directly interacts with hACE2, many research groups have been actively working to discover biomolecules such as antibodies 8-13 and aptamers 14-18, which effectively block the RBD/hACE2 interaction. In addition, a variety of experimental and computational studies have suggested that peptides, small molecules, nanobodies, and other molecular tools could be potentially useful strategies to stop the viral infection via inhibition of the spike-protein/hACE2 interactions 19-47.

Aptamers, also called chemical antibodies, are single-stranded oligonucleotides, which can fold into complex 3D structures, enabling them to bind, through non-covalent interactions, to a large variety of targets such as proteins, nucleic acids, small molecules, or cells 48-50. Aptamers are selected from a large pool of random sequences through an iterative selection process called Systematic Evolution of Ligands by Exponential Enrichment (SELEX) 51, 52. Production of DNA aptamers is significantly more cost-effective than making antibodies, and they can be manufactured using routine chemical synthesis. In comparison to antibodies, aptamers have lower immunogenicity and toxicity 53-57. However, to kill the malignant cells, the selective cytotoxic properties could be imparted to aptamers by conjugating the suitable cytotoxic drugs 58-61. The structural stability of the aptamer and aptamer-target complex is usually considered to be responsible for overcoming the limitations of aptamer-based therapeutics 62-67. The lower chemical and biological stability and the limited chemical space imposed by the native (deoxy)ribonucleotides have restricted the use of aptamers for therapeutic applications 68, 69. However, the limitations can be overcome by the construction of nucleic acid mimics (NAM) aptamers, which comprise the chemical modifications on the heterocyclic base or sugar-phosphate backbone of native nucleic acids 65, 70-86.

Several aptamers have been reported to block the spike-protein/hACE2 interaction 14-18, 87-93. Almost all of these aptamers specifically target RBD, except the SP6 aptamer 88. SP6 does not block the spike-protein/hACE2 interaction, but it reduces pseudovirus infection by interfering with the post-binding process of the pseudovirus to cells via an unclarified mechanism 88. Hence, it is of interest to discover the anti-S2 aptamers which may offer novel strategies for treatment against viral infection. In addition, an anti-S2 aptamer can be conjugated with an anti-S1 aptamer to construct a fusion aptamer that can bind to the spike-protein at two different sites. This may further enhance the inhibition efficacy in blocking the spike-protein/hACE2 interaction, since the fusion aptamer has a larger steric hindrance compared with each mono aptamer.

To accomplish the construction of the hetero-bivalent aptamer 94-98, we performed DNA SELEX on the wild-type (WT) S2-protein and obtained several anti-S2 aptamers, including predominant aptamers S2A1 and S2A2. Both original aptamers and their truncated offspring aptamers bind to the S2- or spike-protein with high affinity and specificity. More interestingly, these anti-S2-aptamers can block the spike-protein/hACE2 interaction, suggesting an RBD independent neutralization mechanism. To further improve the inhibition efficacy, we constructed a fusion aptamer by linking one anti-S2-aptamer (S2A2C1) with one anti-S1B-aptamer (S1B6C3) 16. The fusion probe, through an RBD-dependent approach, binds to the spike-protein at two different sites and shows largely enhanced inhibition efficacy to prevent the spike-protein binding to hACE2. This includes the WT, Delta, and Omicron variant spike-proteins. In short, we demonstrated an RBD-independent approach (by anti-S2-aptamers) and an RBD-dependent approach (by fusion aptamers) to block the spike-protein/hACE2 interactions.

## Result and Discussion

**Figure 2:**
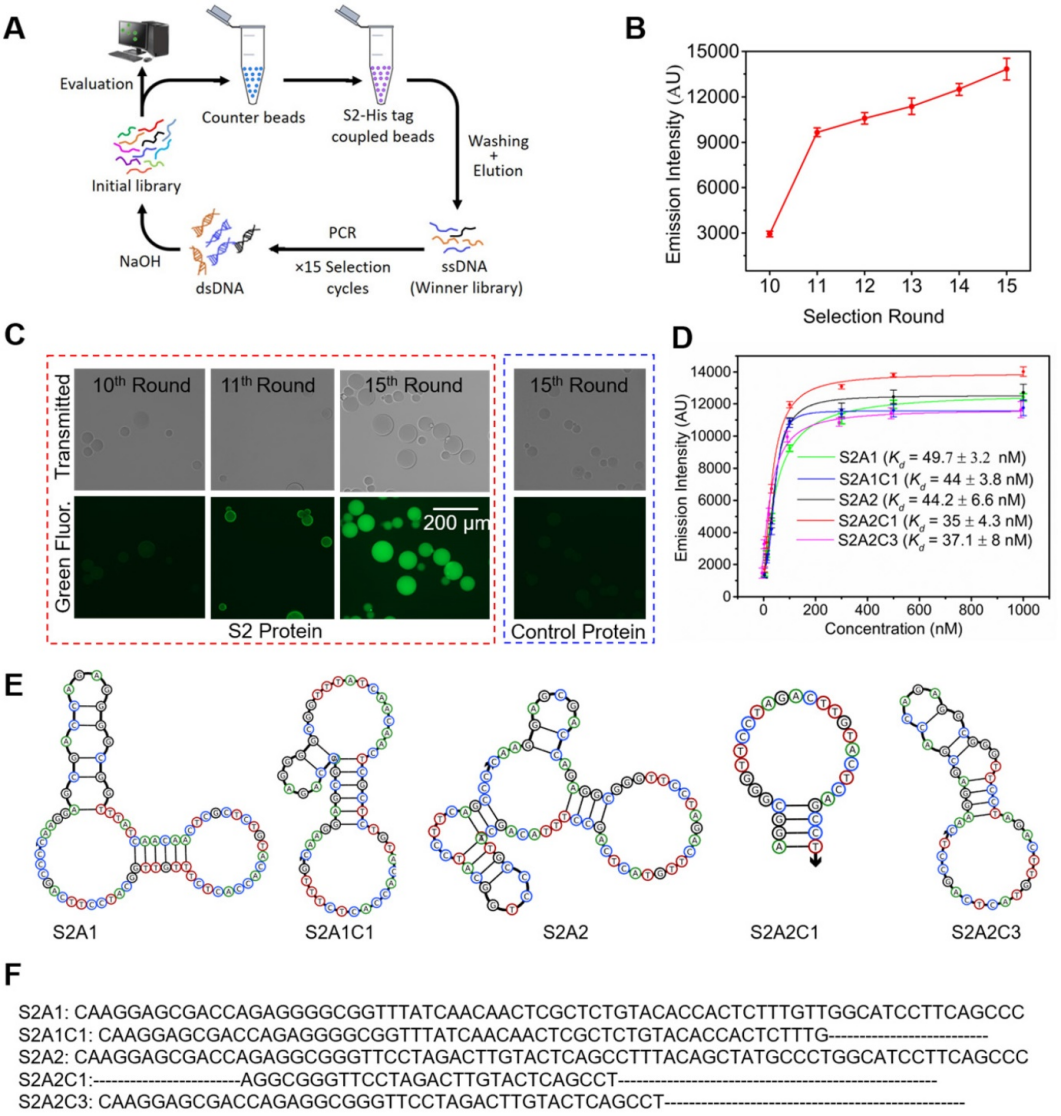
** Selection and characterization of anti-S2 DNA aptamers. (A)** SELEX scheme for the selection of aptamers against the S2-domain of the WT spike-protein. **(B)** Enrichment of the binding capability of the ssDNA library over various selection rounds. The fluorescence emission was measured with a Clariostar microplate reader at *λ_max_* = 520 nm. **(C)** Fluorescence imaging of S2-protein/Ni-NTA resin beads in the presence of the 6-FAM-labeled library was obtained from the 10^th^, 11^th^, and 15^th^ rounds (encircled red dots). This shows that the S2-domain-specific pools are satisfactorily enriched after the 15^th^ round of selection. The complex of the control protein and Ni-NTA resin beads does not produce a fluorescence signal in the presence of the 6-FAM-labeled library obtained from the 15^th^ round of selection. Both the transmitted light (top panel) and green fluorescence channel (bottom panel) images were collected using a digital inverted fluorescence microscope (Invitrogen EVOS FL). The scale bar represents 200 µm. **(D)** Binding affinities (*K_d_*) of the original and truncated anti-S2 aptamers. **(E)** Secondary structures of anti-S2 aptamers were obtained using NUPACK. **(F)** Nucleotide sequences of the anti-S2 aptamers. The aptamers S2A1 and S2A2 are originally obtained from the SELEX, while S2A1C1, S2A2C1, and S2A2C3 are the optimized truncated aptamers obtained from the S2A1 and S2A2 aptamers respectively by the deletion of redundant nucleotides. The nucleotides removed in the optimized aptamers are represented by dotted lines.

**Selection of the S2 specific DNA library, and sequencing analysis.** We performed fifteen cycles of selection against the SARS-CoV-2 WT S2-protein (Figure 2A) using an ssDNA library encompassing a 40-nt randomized region (Table S1). For the initial selection cycle, we used 3 nmol of the DNA library, which provided wide sequence diversity with ∼10^15^ unique DNA molecules. The His-tagged S2-protein was immobilized on nickel nitrilotriacetic acid (Ni-NTA) magnetic beads and incubated with the DNA library in each selection cycle. After washing by selection buffer, the DNA bound to the beads was eluted and subjected to PCR amplification (Figure S1). A counter selection step, starting from the 3^rd^ round, was introduced in each alternative round to remove unspecific DNA binders, using unembellished Ni-NTA beads (Figure 2A). Iterative cycles of selection while reducing the amount of protein and DNA, as well as the incubation time, yielded the enrichment of a specific pool against the S2-protein (Figure 2B, 2C). The details of each selection cycle are listed in Table S2. To monitor and assess the enrichment of the specific binding between the DNA library and the S2-protein over the selection rounds, we generated 6-FAM- labeled ssDNA from different SELEX rounds (from the 10^th^ to the 15^th^ rounds) using a 6-FAM-labeled reverse primer. As shown in Figure 2B, these libraries were incubated with the S2-protein, which was pre-immobilized on magnetic streptavidin beads. After washing, the 6-FAM-labeled libraries were eluted, and their fluorescence intensities were recorded using a Clariostar plate reader. As shown in Figure 2B, the fluorescence signal significantly increased from the 10^th^ to 11^th^ round, and consistently increased to its maximum at the 15^th^ round. In addition, we also determined the specific enrichment using fluorescence microscopy (Figure 2C). The His-tagged S2-protein was immobilized on a Ni-NTA resin and incubated with 6-FAM labeled ssDNAs. After washing, the resin was subjected to fluorescence imaging. As shown in Figure 2C, the pool from the 15^th^ round displayed a much stronger fluorescent signal than that from the 10^th^ or 11^th^ round, Furthermore, the 6-FAM-labeled ssDNA from the 15^th^ round did not bind to the control His-tagged protein immobilized on the Ni-NTA resin (Figure 2C right). This implies that there is high binding specificity of the ssDNA library from the 15^th^ round toward the S2-protein. The above data indicate that after 15 rounds of SELEX, we have obtained a promising aptamer candidate pool with good binding ability against the S2-protein. For this reason, the enriched aptamer candidate pool from the 15^th^ round was cloned using a TOPO TA Cloning® Kit. The product of recombination was used to transform *E. coli* component cells and random colonies were sequenced. We obtained a total of 8 aptamer candidates, whose sequences are given in Table S1. We observed two major groups of aptamer candidates, in which the sequence of S2A1 (5'- CAAGGAGCGACCAGAGGGGCGGTTTATCAACAACTCGCTCTGTACACCACTCTTTGTTGGCATCCTTCAGC CC-3') occupies 20 % of the whole sequencing data, and the sequence of S2A2 (5'-CAAGGAGCGACCAGAGGCGGGTTCCTAGACTTGTACTCAGCCTTTACAGCTATGCCCTGGCATCCTTCAGCCC-3') occupies 57.5 %.

**Characterization of binding affinities for anti-S2 aptamers.** The aptamer candidates S2A1 and S2A2 were synthesized by and received from the Integrated DNA Technology (IDT). Their sequences and secondary structure information are provided in Figures 2E and 2F. The secondary structures of the aptamers were predicted using NUPACK. To reduce the cost of synthesis and other complications that might arise using a longer nucleotide chain, both original aptamers were shortened based on their common sequence and predicted structural analysis, by removing the redundant nucleotides from the 3', 5', or both ends. Three truncated aptamers: S2A1C1, S2A2C1, and S2A2C3 were generated (Figures 2E and 2F). The equilibrium dissociation constants (*K_d_*) of all candidates were measured via a streptavidin bead-based fluorescence assay, in which we measured the response of the fluorescence emission intensity as a function of various concentrations of the 6-FAM-labeled aptamer (3, 10, 30, 100, 300, 1000 nM) bound with 5 pmol of the S2-protein (Figure S2A). As shown in Figure 2D, S2A1 and S2A2 bind the S2 protein with similar binding affinities, *K_d_* = 49.7 ± 3.2 and 44.2 ± 6.6 nM, respectively. We found that three truncated aptamers S2A1C1, S2A2C1, and S2A2C3 show a slightly better binding affinity toward the S2-protein than their parent aptamers (Figure 2D). Among all anti-S2 aptamers, S2A2C1 has the shortest sequence, with a hairpin structure, and also has the best binding affinity toward the S2-protein (*K_d_* = 35 ± 4.3 nM).

**Figure 3:**
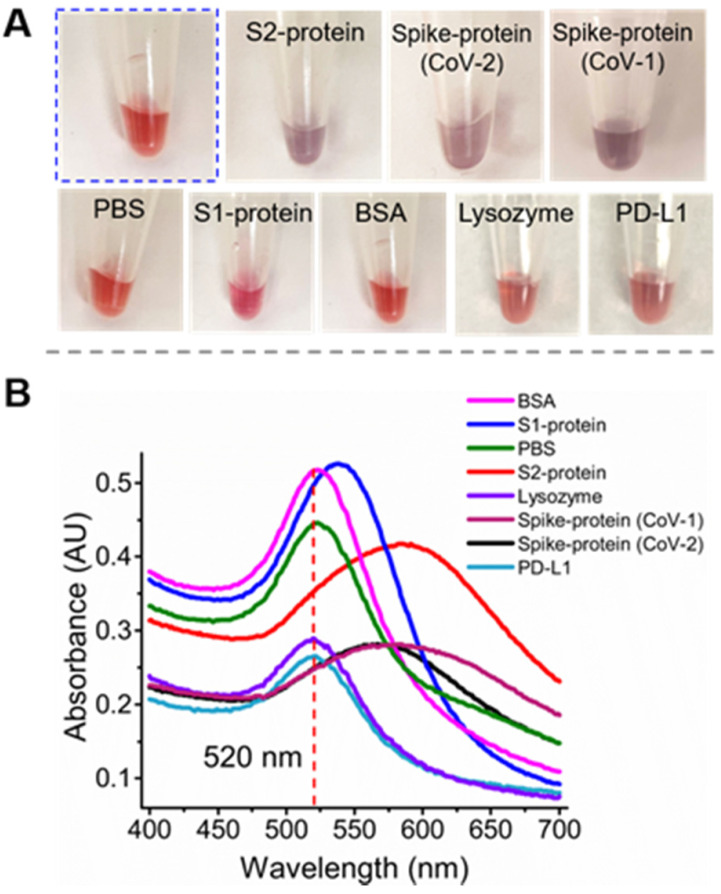
** Specificity test for the anti-S2 aptamer S2A2C1 using an AuNPs-based colorimetric assay. (A)** The addition of PBS solution or 250 nM solution of non-specific proteins such as S1-protein, BSA, lysozyme, or PD-L1 to the colloids of AuNPs, 1.5 M NaCl, and 250 nM S2A2C1 aptamer does not impart specific interaction with S2A2C1 aptamer, hence the red-wine color persists for more than 48 h; When 250 nM solution of S2-, SARS-CoV-2 spike-protein (CoV-2), or SARS-CoV-1 spike-protein (CoV-1) was added, it shows the specific interaction to the aptamer present in the AuNPs colloids; hence the purple color was observed within 5 minutes of addition. **(B)** UV-Vis absorption spectra of AuNP solutions containing the 250 nM of S2A2C1 aptamer after addition of 250 nM of spike-protein, S2-protein, S1-protein, BSA, PBS-buffer, Lysozyme, and PD-L1. The redshift indicates the formation of AuNPs aggregation.

**Figure 4:**
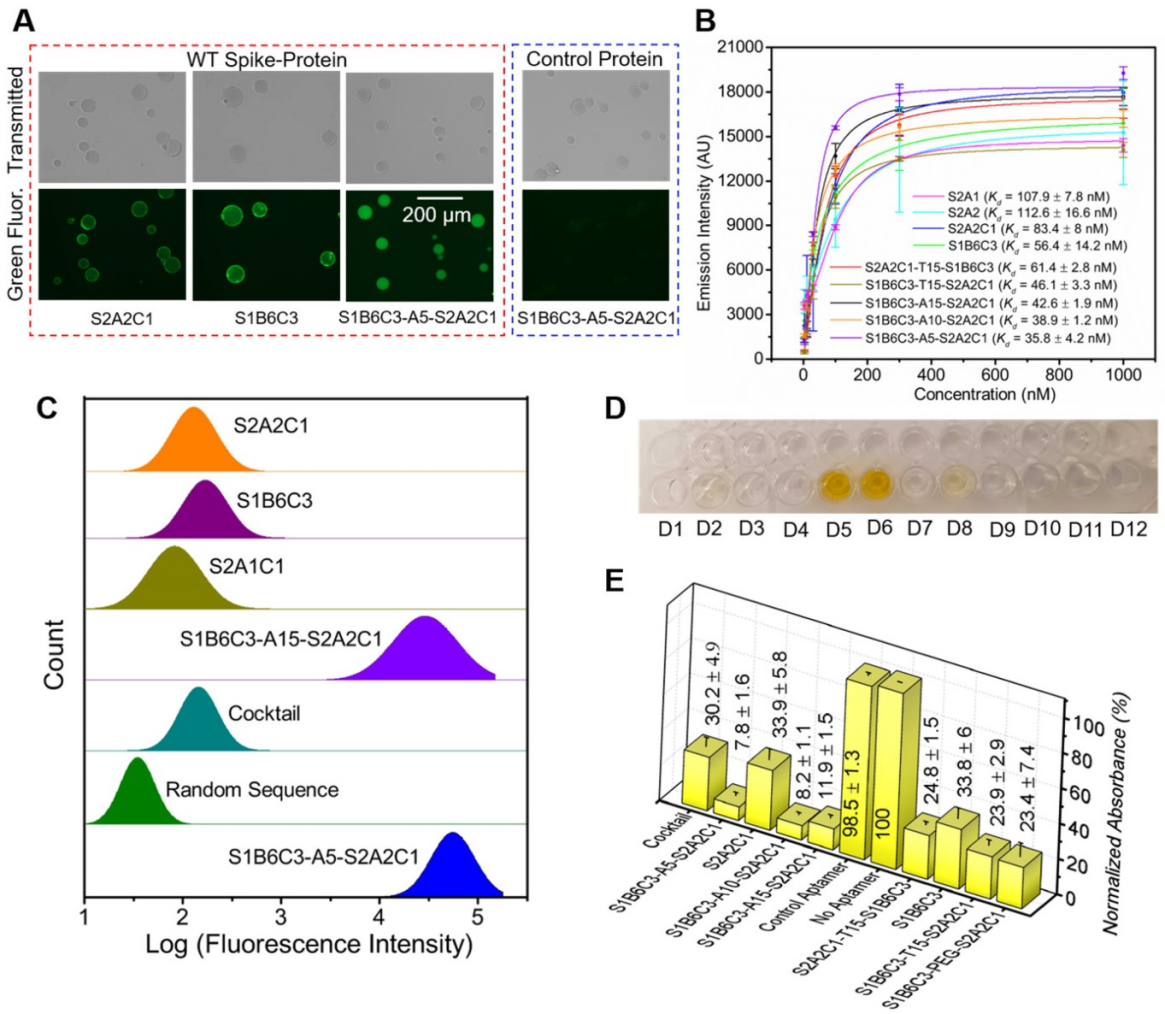
** Determination of the binding affinities and inhibition efficacies of aptamers to the WT SARS-CoV-2 spike-protein. (A)** Fluorescence imaging of Ni-NTA resin beads in the presence of the His-tagged WT spike-protein and FAM-labeled mono and fusion aptamers. The protein and 6-FAM-labeled aptamers are used in the same concentration of 100 nM. The rightmost image shows that Ni-NTA resin beads do not give a fluorescence signal in the presence of the unspecific control protein and S1B6C3-A5-S2A2C1, indicating the aptamer does not bind to the control protein. The scale bar represents 200 µm. **(B)** Binding affinities (*K_d_*) of mono and fusion aptamers against the His-tagged WT spike-protein. **(C)** Flow cytometry measurements of the green fluorescence emission from magnetic beads in the presence of the WT spike-protein and various aptamers. The WT spike-protein and 6-FAM-labeled aptamers are used in the same concentration of 500 nM. The library with random sequences showed the minimum, and S1B6C3-A5-S2A1C1 fusion aptamers showed the maximum binding potentials, respectively, to the WT spike-protein. **(D)** Color of the final product in ELISA tests corresponding to various aptamers: D1 = S1B6C3-A5-S2A2C1, D2 = S2A2C1, D3 = S1B6C3-A10-S2A2C1, D4 = S1B6C3-A15-S2A2C1, D5 = aptamer control, D6 = no aptamer, D7 = S2A2C1-T15-S1B6C3, D8 = S1B6C3, D9 = S1B6C3-T15-S2A2C1, D10 = S1B6C3-PEG-S2A2C1, D11 =TMB only, D12 = SELEX buffer. An intense yellow color was obtained due to HRP-mediated oxidation of the TMB when an unspecific (control) or no aptamer was added. **(E)** Normalized relative absorbances of the final ELISA products corresponding to neutralization efficacies of the various aptamers. The absorbance is inversely related to the neutralization efficacy of the aptamer.

Next, we asked whether these anti-S2 aptamers can still recognize the S2-domain in the whole spike-protein, since the S2-protein may undergo conformational changes when it is co-expressed with the S1-protein. To answer this question, we measured the binding potential of anti-S2 aptamer S2A2C1 against the whole spike-protein (Figure 3). The data show that S2A2C1 can recognize either the S2-protein or the S2 domain in the spike-protein. In addition, we also measured the *K_d_* values for S2A1, S2A2, and S2A2C1 against the spike-protein. As shown in Figures 2D and 4B, both original aptamers and the optimized S2A2C1 were observed to bind well to spike-protein, with moderately higher binding *K_d_* values compared to S2-protein. Since the S2A2C1 aptamer showed the best binding to the spike- and S2-proteins, we used it to study the binding specificity.

**Characterization of the binding specificity of S2A2C1.** To validate the binding specificity of our most desirable truncated aptamer S2A2C1 to S2-protein, we performed the well-established gold nanoparticles (AuNPs)-based colorimetric assay 39, 99, 100. We used AuNPs-based colorimetric assay which confirmed that the S2A2C1 aptamer does not bind the S1-protein and only binds to the S2-protein or S2 domain in the spike-protein of SARS-CoV-2. The AuNPs colorimetric assay, as demonstrated in Figure S2C, is based on the function of negatively charged DNA molecules to prevent the AuNPs aggregation. The detailed mechanism and kinetics of the interaction between aptamer and AuNPs that prevents AuNPs from aggregating have been extensively characterized by Nelson and Rothberg 101. Briefly, when a specific target of the DNA aptamer in AuNPs colloids is added, the aggregation starts, which then changes the color of AuNPs from wine-red to blue or purple. In the presence of the S2A2C1 aptamer, the AuNPs persist red color (Figure 3A, encircled with blue dots). When the S2- or spike-protein of SARS-CoV-2 was added into the colloids of AuNPs, NaCl, and the S2A2C1 aptamer, the protein was preferably bound to the S2A2C1 aptamer, activating the AuNPs aggregation. The wine-red color of the AuNPs colloids was intact for more than 48 hours in 1.5 M NaCl and 250 nM S2A2C1 aptamer. However, it dramatically changed into blue or purple within 5 minutes of adding 250 nM of the S2- or spike-protein (Figures 3A, and S2C). Besides visual color change, UV-Vis measurement was also employed to measure the effect of various targets on the colloids of AuNPs, S2A2C1 and NaCl (Figure 3B). The S2-, and SARS-CoV-2 spike-protein caused the significant redshift to the characteristic peak of AuNPs colloids located at 520 nm. However, nonspecific targets such as the S1-protein, BSA, lysozyme, or PD-L1 did not cause the obvious redshift to the characteristic peak (Figure 3B). This experimental evidence states that S2A2C1 specifically binds to the S2-domain regardless of whether it is isolated or co-expressed in the whole spike-protein. Since an aptamer maintains its binding specificity toward the target as long as the target preserves its identity, we next asked whether S2A2C1 can also recognize the S2-subunit of the SARS-CoV-1 spike-protein. The protein sequence alignment showed that the spike-protein S2-subunits of SARS-CoV-1 and SARS-CoV-2 are highly conserved (about 93.1 % conserved residues) 102, 103. Based on this fact, we assumed that the S2A2C1 aptamer should also be able to recognize the SARS-CoV-1 spike-protein. To examine this, we performed the colorimetric assay by employing the SARS-CoV-1 spike-protein as a new target. The result showed that the aptamer S2A2C1 can also bind to the SARS-CoV-1 spike-protein (Figure 3), indicating our anti-S2 aptamer, S2A2C1, can recognize both spike-proteins of SARS-CoV-1 and SARS-CoV-2.

In addition to the redshift of the absorbance peak of AuNPs, the ratios of A520/A620 can be used to define the specificity of the interaction between the proteins and the aptamer 104. It should be mentioned that the A520/A650 ratio is not calibrated to the percent bound. As shown in Figure S2D, the A520/A650 values are greater than 3 for S1, BSA, and buffer, indicating that they have negligible binding to the S2A2C1 aptamer. The A520/A650 values are smaller than 1 for the S2-, and SARS-CoV-2 spike-protein; it refers to being the specific binders to S2A2C1. In summary, the aptamer S2A2C1 can specifically bind to the S2-protein, but not to the S1-protein (RBD).

**Anti-S2 aptamer S2A2C1 inhibits the spike-protein/hACE2 interaction via an RBD-independent approach.** The aptamer S2A2C1 was obtained by SELEX on the S2-protein, and we also confirmed that it specifically binds to the S2, but not to the S1-protein. Following our initial plan, we decided to construct a fusion aptamer, consisting of S2A2C1, a linker, and an anti-S1 aptamer S1B6C3 developed by the Yang group 16. The S1B6C3 aptamer (5'-CGCAGCACCCAAGAACAAGGACTGCTTAGGATTGCGATA-GGTTCGG-3') was selected against RBD and can efficiently block the spike-protein/hACE2 interaction. Before constructing the fusion aptamer, we measured the binding affinity of S1B6C3 toward the spike-protein, with *K_d_* = 56.4 ± 14.2 nM, which is lower than that of S2A2C1 on spike-protein, *K_d_* = 83.4 ± 8 nM, as shown in Figure 4B. Flow cytometry measurements also demonstrate that the 6-FAM-labeled S2A2C1 and S1B6C3 virtually have similar binding potential toward the WT spike-protein (Figure 4C). Next, we used an Enzyme-Linked Immunosorbent Assay (ELISA) to examine how both aptamers inhibit the spike-protein/hACE2 interaction. Briefly, the plate wells were coated with non-His-tagged hACE2 and then blocked by BSA to avoid any nonspecific binding. After washing, the His-tagged spike-protein was added which can be recognized by an HRP anti-His-tag antibody with the substrate TMB (Figure S2E). This gives a strong absorbance at 450 nm after adding sulfuric acid (Figure 4D, well D6). The normalized absorbance is proportional to the amount of target protein present. In the presence of an aptamer such as S1B6C3, which can effectively neutralize spike-protein and prevent its binding to hACE2, the His-tagged spike-protein will be removed during washing. Consequently, HRP cannot generate correspondingly strong signals. As shown in Figures 4D (well D8) and 4E, S1B6C3 inhibited 66% of spike-protein to bind to hACE2, as expected. Interestingly, S2A2C1, as an anti-S2 aptamer, also inhibited 66% of spike-protein binding to hACE2, as shown in Figures 4D (well D2) and 4E. At present, this RBD-independent mechanism is unknown. Most likely once S2A2C1 binds to the S2-protein, it induces an allosteric effect on the S1-protein, affecting its binding with hACE2. Further study on the complex structure may provide more information. The control aptamer cannot prevent the spike-protein/hACE2 interaction (Figure 4D, well D5 and 4E), indicating both S1B6C3 and S2A2C1 specifically block spike-protein.

**Design of fusion aptamers and characterization of their binding affinity and inhibition efficacy against WT spike-protein.** Next, we constructed fusion aptamers using aptamers S1B6C3 and S2A2C1 with various linkers such as polyethylene glycol (PEG), T25, T15, A15, A10, and A5 (Table S3). We expect that these fusion probes can bind to the spike-protein at two sites, offering improved binding affinity and inhibition efficacy. First, we generated four fusion aptamers: S2A2C1-T25-S1B6C3, S2A2C1-T15-S1B6C3, S1B6C3-T25-S2A2C1, and S1B6C3-T15-S2A2C1; all have a long polyT linker (25- or 15-mer), but the constituent mono aptamers reside in a different orientation, since this direction may affect the function of the corresponding fusion aptamer. We first measured the inhibition efficacies for the four fusion aptamers and observed that all of them have very similar inhibition efficacies to block the spike-protein/hACE2 interaction (Figure S3B). This indicates that there is almost no difference in using T25 vs. T15 linkers. To save the cost of synthesis, we used T15 as the linker and measured the binding affinities of aptamers S2A2C1-T15-S1B6C3 and S1B6C3-T15-S2A2C1 to decide which orientation is better. As shown in Figure 4B, S1B6C3-T15-S2A2C1 (*K_d_* = 46.1 ± 3.3 nM) is superior to S2A2C1-T15-S1B6C3 (*K_d_* = 61.4 ± 2.8 nM) in recognizing the WT spike-protein. Therefore, we set S1B6C3 at the 5' end for subsequent fusion aptamer design. Next, we tested the change of the linker T15 to A15 or a PEG linker, which has a similar length compared to the former two linkers. Although this change does not significantly affect the predicted secondary structures of corresponding fusion aptamers (Figure S3C), it may still influence their functions. As shown in Figure 4E, S1B6C3-A15-S2A2C1 has a better inhibition efficacy (with its presence, only 12 % of the spike-protein can bind to hACE2) than that of S1B6C3-T15-S2A2C1 (24 %) or S1B6C3-PEG-S2A2C1 (23 %), encouraging us to continue using a PolyA linker. Finally, we tried to further shorten the linker length, thoroughly measuring the improvement in binding affinity and inhibition efficacy by comparing A15, A10, and A5 linkers. We conclude that S1B6C3-A5-S2A2C1 is the most desirable fusion aptamer (Figure 4B, 4C, 4E, and Figure S4), which has approximately 2.3 times better binding affinity (*K_d_* = 35.8 ± 4.2 nM) in comparison with constituent mono aptamers. It should be mentioned that we also compared S1B6C3-A5-S2A2C1 with the aptamer cocktail (a mixture of S1B6C3 and S2A2C1 in a 1:1 molar ratio). The former is a single DNA molecule and the latter contains two molecules. In the presence of S1B6C3-A5-S2A1C1, only 8 % WT spike-protein can bind to hACE2, but with the same molar concentration of both mono aptamers in the cocktail, more than 30 % WT spike-protein can still bind to hACE2 (Figures 4D, 4E, and S4). These results show that the fusion aptamer, as a single molecular probe, enhances inhibition efficacy in comparison to the cocktail, to block spike-protein binding to hACE2. All the above data indicate a single molecular fusion aptamer is superior to cocktails of mono aptamers in binding to WT spike-protein and inhibiting the spike-protein/hACE2 interactions. We will continue to use the optimized fusion aptamer S1B6C3-A5-S2A2C1 for subsequent experiments.

**Figure 5:**
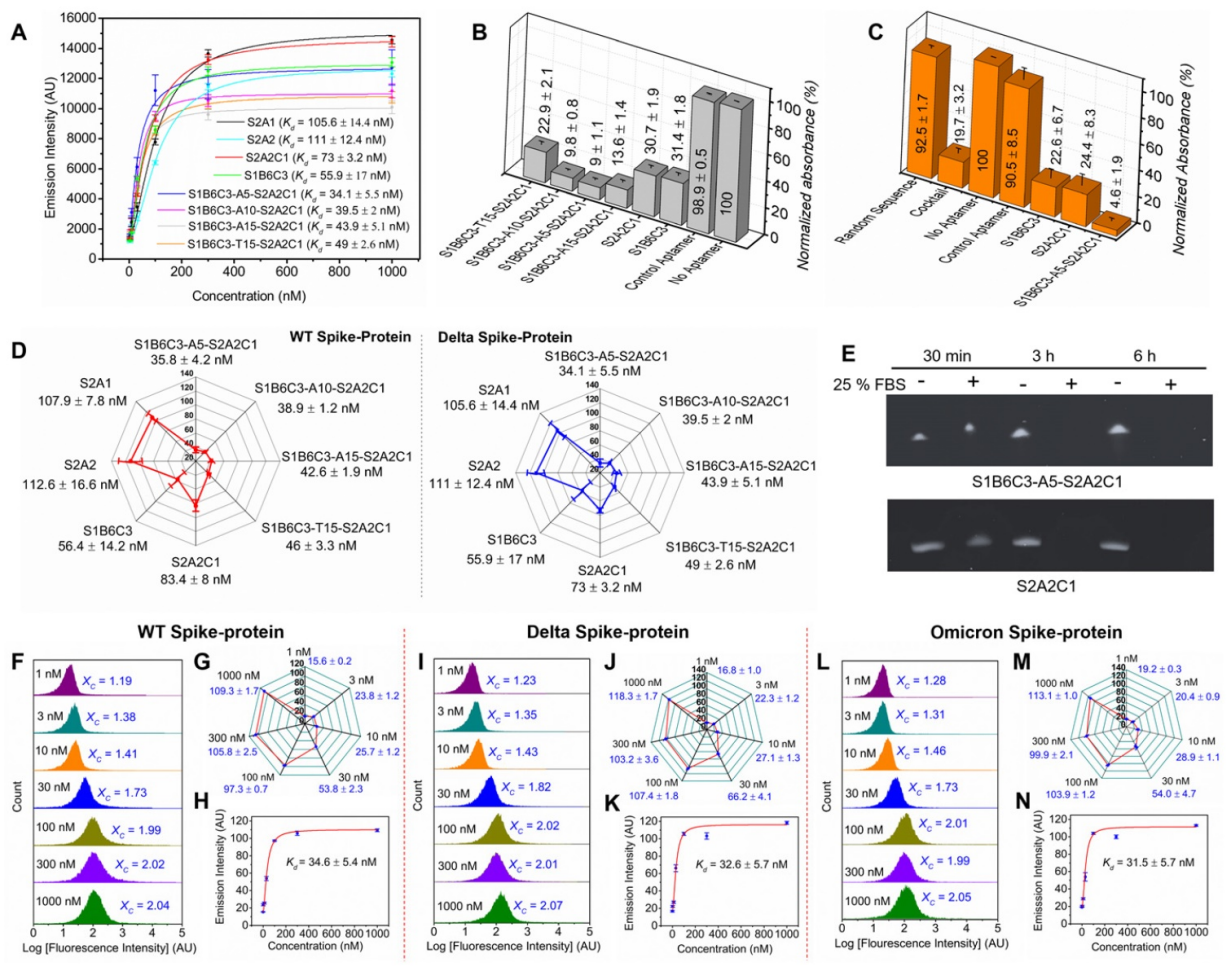
** Binding affinities, inhibition efficacies, and stability of the mono and fusion aptamers. (A)** Binding affinity (*K_d_*) of the aptamers with the Delta variant spike-protein. **(B)** Inhibition efficacies of aptamers or the control aptamer against Delta spike-protein by employing the competitive ELISA experiment. Relative normalized absorbances obtained from the colorful product in the presence of aptamer is inversely proportion to its inhibition efficacies. **(C)** Inhibition efficacies of the aptamers against the Omicron spike-protein. **(D)** The comparison of the binding affinities of aptamers toward the WT and Delta spike-protein reveals that the aptamers bind to both spike-proteins with comparable affinities. **(E)** The stability assessment of the S2A2C1 and S1B6C3-A5-S2A2C1 in 25 % FBS. The 4 ng/μL aptamer samples were prepared in nuclease free water and incubated at 37 °C for various time in the absence (-) or presence (+) of the 25 % FBS solution. the aptamer samples showed the noticeable digestion in 30 min and complete digestion within 3 h by the action of 25 % FBS. The stability of the aptamers in the biological environment maybe improved by the construction of nucleic acid mimics (NAM) aptamers, which comprise the chemical modifications on the heterocyclic base or sugar-phosphate backbone of native nucleic acids. **(F-N)** The determination of *K_d_* of the 6-FAM-labeled S1B6C3-A5-S2A2C1 aptamer by using the flow cytometry approach. The flow cytometry measurement of the aptamer bound to (F-H) WT spike-protein, (I-K) Delta spike-protein, and (L-N) Omicron spike-protein. Figures F, I, and L are the histograms obtained from the flow cytometry measurement to determine the mean green fluorescence emission intensity (*X_C_*) of 6-FAM-labeled S1B6C3-A5-S2A2C1 aptamer bound to the complex of Ni-NTA beads and the His-tagged spike-protein. Each histogram was constructed by the integration of 3 trials of experiments that contain a total of 15,000 flow events (5000 events from a trial). Figures G, J, and M show the increment of *X_C_* value as a function of 6-FAM aptamer concentration that bound to virtually the same quantity of spike-protein and Ni-NTA bead complex. The shorter error bars indicate the high reproducibility of *X_C_* values for various trials. Figures H, K, and N represent the *K_d_* of the 6-FAM labeled S1B6C3-A5-S2A2C1 aptamer against various spike-proteins. The *K_d_* values were determined by intensity vs concentration plot using the Origin software.

**Aptamers show decent binding and inhibition efficacy to the Delta and Omicron spike-proteins.** Based on CDC reports, the Delta and Omicron variants have a few mutations in the gene encoding the SARS-CoV-2 spike-protein. Most mutations are located on the S1-protein, with only several on the S2-protein, indicating that the S2-protein is highly conserved among variants 102, 103, 105. To examine whether S2A2C1 and the fusion aptamers can still inhibit these mutated spike-proteins recognizing hACE2, we first performed experiments to determine the binding affinity and inhibition efficacy of mono, cocktail, and the fusion aptamers against the Delta spike-protein. Figure 5A shows the *K_d_* values of various aptamers against Delta spike-protein. Figures 5B and 5C show the inhibition efficacies of various aptamers against the Delta and Omicron spike-protein, respectively. Comparing the *K_d_* values of various aptamers with the WT and Delta spike-proteins, as shown in Figure 5D, we observed that all tested aptamers have very similar binding affinities on both WT and Delta spike-proteins, indicating that mutated amino acids on the Delta spike-protein do not have much influence to the binding between these aptamers and the spike-protein. We, therefore, speculate that these aptamers should also be able to efficiently block the Delta spike-protein/hACE2 interaction. This hypothesis is supported by the data shown in Figures 5B, S5, and S6. The results show that in the presence of S2A2C1, 31 % Delta spike-protein can bind to hACE2, and this value decreases to 9 % when S1B6C3-A5-S2A2C1 was used.

Next, we further discussed how S1B6C3-A5-S2A2C1 is better than mono and other fusion aptamers in inhibiting the Omicron spike-protein/hACE2 interaction. As shown in Figures 5C and S6, in the presence of S1B6C3-A5-S2A2C1, only 5 % Omicron spike-protein can still bind to hACE2. For S2A2C1 the residual binding is 24 %, and for the aptamer cocktail, it is 20 %. Our results conclude that S2A2C1 and the fusion aptamer S1B6C3-A5-S2A2C1 bind to both Delta and Omicron spike-proteins irrespective of their mutated residues. It was reported that the variants do not cause large conformational changes in the SARS-CoV-2 spike-protein 106-109**,** which could be the reason why our anti-WT aptamers can still neutralize the Delta and Omicron spike-proteins, regardless of the mutant residues. Results from overall binding affinity and inhibition assays demonstrate that the fusion aptamer S1B6C3-A5-S2A2C1 is a potential tool to prevent SARS-CoV-2 and its variants from infecting cells.

**Stability of the mono and fusion aptamers in serum.** Some (deoxy)ribonucleic acid aptamers have certain stability without additional chemical modifications, but it requires some structural characteristics. For instance, aptamers having the circular or G-quadruplex structures showed significant stability in serum or blood stream 16, 91, 110. Sun et. al. has reported the bivalent circular aptamers (cb-CoV2-6C3) that retain sequence integrity in the incubation with 95 % human plasma for 12 h and cell media (containing 10% FBS) for 48 h 16. However, the structurally unmodified monovalent aptamer (CoV2-6C3) was easily degraded after incubation with cell media (containing 10% FBS) within 6 h. Similarly, Yang et. al., and Gupta et. al. have independently discovered the Guanine(G)-rich aptamers which can impart stability due to the thermodynamically stable G-quadruplex structures 91, 110. Since our mono and fusion aptamers do not maintain those structures, we decided to check their stabilities in serum.

The S2A2C1 aptamer, 4 μg/mL, was treated with 25 % fetal bovine serum (FBS) at 37 °C for 30 min, 3 h, and 6 h. The stability of the aptamer was evaluated by the denaturing urea polyacrylamide gel electrophoresis (PAGE). The gel data showed that S2A2C1 aptamer has noticeable digestion by serum nucleases in 30 min, and complete digestion in 3 h (Figure 5E, top panel). The control experiment was carried out by incubating aptamer at 37 °C in nuclease free water, which shows that S2A2C1 remains intact for more than 6 h in water. The same procedure was followed to measure the stability of 4 μg/mL S1B6C3-A5-A2S2C1 aptamer in 25 % FBS or nuclease free water. The gel data show that S1B6C3-A5-S2A2C1 is relatively stable in serum in 30 min, and completely digested by 25 % FBS in 3 h (Figure 5E, bottom panel). Above data indicate that further chemical modifications may be required to improve the stabilities of our aptamers when using in serum or blood stream. For example, they can be modified into the nucleic acid mimics (NAM) aptamers, which is accomplished by the chemical modifications on the heterocyclic base or sugar-phosphate backbone of the native nucleic acids 65, 70-86.

**Flow cytometry approach to confirm the binding affinity of the S1B6C3-A5-S2A2C1 aptamer against the WT, Delta, and Omicron spike-proteins**. In addition to the *K_d_* determination using a Clariostar microplate reader, we have also used the flow cytometer to determine the *K_d_* values of our most desirable fusion aptamer S1B6C3-A5-S2A2C1 with the WT, Delta, and Omicron spike-proteins (Figures 5F-N). To calculate the *K_d_* values, we first determined the average intensity of the green fluorescence emission (*X_c_*) from the integrated histogram of the flow cytometry measurements (Figures 5F, I, and L). The integrated histogram contains three trials of flow cytometry measurement and together 15,000 flow events. The flow cytometry measurement was performed for the 1, 3, 10, 30, 100, 300, and 1000 nM concentration of the aptamer incubated in 200 nM spike-proteins coupled with Ni-NTA magnetic beads. The fluorescence emission increases steeply as a function of aptamer concentration when the concentration is increased from 1-100 nM, but emission does not increase noticeably when the concentration is increased beyond the range of 100 nM (Figures 5G, J, and M). The *K_d_* of S1B6C3-A5-S2A2C1 aptamer against spike-protein was determined by plotting the graph of average fluorescence (*X_c_*) versus aptamer concentration using Origin software (Figures 5H, K, and N). The binding affinity (*K_d_*) of the S1B6C3-A5-S2A2C1 aptamer against the WT, Delta, and Omicron determined by flow cytometry are 36.4 ± 5.4, 32.6 ± 5.7, and 31.5 ± 5.7 nM, respectively, which are consistent with those determined using a Clariostar microplate reader. The comparison of the *K_d_* values measured by the Clariostar microplate reader and flow cytometer is summarized in Table S4.

## Conclusion

We discovered anti-S2 aptamers by exploiting DNA SELEX methods on the WT S2-protein. Redundant nucleotides from the original aptamers were removed to obtain truncated aptamers with good binding specificity and affinity toward the S2-protein. As the most desirable anti-S2 aptamer, S2A2C1 showed the maximum binding affinity, and more importantly, it also showed the virtuous efficacy to neutralize the WT spike-protein/hACE2 interaction, suggesting an RBD-independent approach. To further improve its binding and inhibition efficacy, we conjugated the S2A2C1 aptamer with a reported anti-RBD aptamer, S1B6C3, by testing various linkers to construct fusion aptamers, among which S1B6C3-A5-S2A2C1 has the best binding affinity on WT, Delta, and Omicron spike-proteins. It also has the best inhibition efficacy against the WT, Delta, and Omicron spike-protein/hACE2 interaction. Both S2A2C1 and S1B6C3-A5-S2A2C1 maintain high inhibition efficacy in preventing Delta or Omicron spike-protein binding to hACE2, making them well-suited as diagnostic and therapeutic molecular tools against SARS-CoV-2 and its variants. In addition, the past 20 years have witnessed three fatal and well-documented zoonotic coronaviruses (CoVs) outbreaks: SARS-CoV-1 in 2002, MERS-CoV in 2012, and SARS-CoV-2 in late 2019. Although we do not know when the current COVID-19 pandemic will end, ecological reality and current scientific evidence suggest that a new coronavirus may evolve in the forthcoming future. Our approach to discovering aptamer inhibitors targeting the relatively conserved S2-protein, as well as our strategy to design fusion aptamers, can also be used to target any new coronaviruses as they emerge in the near future.

## Experimental Methods and Materials

**Chemical and reagents.** All proteins including hACE2, the WT, and variant SARS-COV-2 spike-proteins used in this work were purchased from Sino Biological and used without further purification. All aptamers and other nucleic acids were obtained from Integrated DNA Technologies, Inc. (www.idtdna.com) as lyophilized powders and were dissolved in nanopure water upon receipt. All chemicals were purchased from Sigma unless mentioned otherwise.

**SELEX procedure.** We performed the DNA-SELEX using the S2-domain of the WT spike-protein as a target. We used an oligonucleotide library obtained from IDT which is composed of 40 random nucleotides flanked by constant primer sequences (Table S1). For the first round of the selection, the 100 pmol of the S2-protein and 1 µL of nickel nitrilotriacetic acid (Ni-NTA) beads (G-bioscience) were diluted into 100 µL of SELEX buffer (PBST-Mg buffer, PBS with 1 mM MgCl_2_, pH 7.4, 0.01 % tween) and incubated at room temperature (RT), rotating for 1 h. Meanwhile, 3 nmol of DNA library was diluted into 100 µL of SELEX buffer and treated at 95 °C for 5 min, on ice (or 4 °C) for 5 min, RT for 5 min, and placed in ice. When 1 h incubation was completed, protein-bead (P-B) complex was washed twice by 200 µL SELEX buffer and mixed with the heat-treated DNA library. 1 µL of 100 mg/mL tRNA and incubated for 1 h at RT with rotation. After incubation, the protein-bead-library (PBL) complex was washed twice with 200 μL SELEX buffer to remove the unspecific library. After washing, the selected library was eluted twice with 30 μL of hot water at 95 °C. The selected library was then amplified by the polymerase chain reaction (PCR). For the first round of selection, the PCR mixture contains 60 µL of the library, 39 µL of nuclease-free water, 100 µL of 2 × PCR solution, and 1 µL of Easy Taq polymerase. The 50 µL of the PCR mixture was loaded into each PCR tube and amplified in the conditions of 2 min at 95 °C; 9 cycles of 45 s at 95 °C; 30 s at 54 °C; 30 s at 72 °C, and 2 min at 72 °C. After completing PCR, all PCR product was collected in a tube. To optimize the PCR cycle number for bulk amplification, 5 µL of the PCR product, 119 µL water, 125 µL of 2×PCR solution, and 1.25 µL Easy Taq polymerase were mixed in a tube and then distributed equally (50 μL) into 5 PCR tubes. Amplification conditions were: 2 min at 95 °C; 3-11 cycles of 45 s at 95 °C; 30 s at 54 °C; 30 s at 72 °C; and extra 2 min at 72 °C. The PCR tubes were taken out in 3, 5, 7, 9, and 11 cycles, respectively, and kept on ice. Then, PCR products were assessed with 2 % agarose gel electrophoresis to determine the suitable number of PCR cycles (X). The suitable number of the PCR cycles provides the right PCR product which was confirmed by a brighter and smear-free band at 73 base pairs (Figure S1). Once the number of suitable PCR cycles (X) was determined, the bulk PCR reaction was run to generate 1 (or 2) mL of PCR mixture (20 µL of the 1st round PCR solution, 475 µL water, 500 µL of 2 x PCR solution, and 5 µL of Easy Taq Polymerase). The PCR amplification conditions were set to be 2 min at 95 °C; X cycles of 45 s at 95 °C; 30 s at 54 °C; 30 s at 72 °C; and an extra 2 min at 72 °C.

After a bulk PCR, 20 μL of neutravidin beads were washed two times by 400 μL of SELEX buffer and incubated with 1 mL of PCR products for 15 minutes rotating at RT. Then. the beads were washed two times with 400 μL of the SELEX buffer. The sense strand was separated from the beads by denaturing in 200 μL of 100 mM NaOH solution for 1 min; the solution was immediately neutralized by 88 µL of 0.2 M HCl. Then the beads were again eluted by 212 µL of SELEX buffer, combined with the previous solution, and centrifuged using a desalting column (3K) at 12000 g for 10 min. The remaining solution in the desalting column was washed two times using 400 µL of the SELEX buffer. The eluted library was quantified by nanodrop, then it was treated at 95 °C for 5 min, ice for 5 min, and RT for 5 min and stored at -20 °C.

For a subsequent round of selection, the 100 pmol DNA library (~2 µg) was incubated with 100 pmol protein (bead complex). The amount of protein and incubation time were consistently decreased in the following selection rounds to increase the selection pressure (Table S2). While the number of washes to the PBL complex was consistently increased to ensure the removal of the unspecific libraries. The bound libraries were eluted two times by 30 µL of hot water at 95 °C. Then, 20 µL from the total 60 µL elution was used for the PCR amplification, and the remaining 40 µL was stored at -20° C. The PCR amplification, purification, desalting, and quantification were similarly followed as explained before. However, after the second SELEX, we introduced the counter selection (CS) in each alternative round. For that, we incubated the ssDNA with the unembellished Ni-NTA magnetic beads, and the unspecific library bound to the magnetic beads was discarded while the specific library present in the supernatant was used to start the next round of selection. The schematic representation of the DNA SELEX is shown in Figure 2A.

**Plasmid preparation for DNA sequencing**. The label-free dsDNA obtained from the 15^th^ selection round was purified using NucleoSpin Gel and PCR Clean-up kit (Ref. # 740609-250) and used as an insert. We used the TOPO TA cloning kit (Ref. # 45-0071) for ligation and transformed *E. coli* component cells using recombinant DNA. The ampicillin-resistant bacterial colonies were cultured on the ampicillin (100 µg/mL)-containing Luria broth (LB) agar plate (following the standard protocol). The bacterial culture was subjected to PCR to assess the correct insert using gel electrophoresis (Figure S7). The plasmid was extracted from the bacterial solution possessing a desirable insert using E.Z.N.A.® Plasmid DNA Mini Kit (Ref. # D6942-01) and sequenced by the Human Genetics Comprehensive Cancer Center DNA Sequencing Facility at the University of Chicago.

**ELISA assay with HRP anti-His-tagged antibody**. The 0.5 µg of hACE2 in 50 μL 0.1 M NaHCO_3_ (pH 8.6) was added to the high binding 96 well plates (Fisher brand Ref. # 12565501) and incubated overnight. The solution was removed and incubated with 100 μL of 5 mg/mL BSA in 0.1 M NaHCO_3_ for 1 h at RT and washed 3 times with 200 μL SELEX buffer containing Tween 20. Then, the 50 μL solution of 100 nM His-tagged spike-protein and the aptamer in SELEX buffer were incubated at RT for 1 h. The plate was then washed six times with 200 μL SELEX buffer to remove the unbound spike-protein. Then, 50 μL of 2000 times diluted anti-His-tagged HRP antibody in SELEX buffer was added, which can bind to remaining His-tagged spike-protein. The wells were incubated at RT for 30 min and washed six times. When the aptamer shows the capacity to block spike-protein/hACE2 interaction, HRP will not persist in the well plate after washing. Finally, 50 μL of TMB substrate solution was added to the well and incubated for 30 minutes at RT. The intense blue color produced in this step shows that the spike-protein has a strong interaction with hACE2. When 2 μL of concentrated sulfuric acid was added to the blue product, the yellow color was formed. The absorbance of the yellow product was measured at *λ_max_* = 450 nm using the Clariostar microplate reader (BMG LABTECH).

**Fluorescence microscope-based binding assay:** The 20 µL of 10× diluted Hispur Ni-NTA resin bead (Ref. # 88221; Thermo scientific) was washed two times with 500 µL of SELEX buffer, resuspended in 50 µL of SELEX buffer, and incubated with 5 pmol of His-tag target protein for 30 minutes at RT with rotation. The resin-protein complex was washed twice with 500 µL of SELEX buffer to remove unbound protein and resuspended with 50 µL of SELEX buffer. The 10 pmol of 6-FAM-labeled ssDNA or aptamer was incubated in the complex of Ni-NTA resin bead and His-tagged protein at RT for 30 min. After washing twice with 500 µL of SELEX buffer, the complex was finally resuspended in 50 µL of SELEX buffer and transferred on a glass slide for the fluorescence imaging. The fluorescence image was collected using both the green fluorescence and transmitted light channels by employing the digital inverted fluorescence microscope (Invitrogen EVOS FL).

**Determination of binding affinity.** The 100 nM His-tagged protein and anti-His tagged biotinylated antibody was incubated at RT for 2 h in 50 μL of SELEX buffer. The complex of the protein and antibody was then incubated with 2 μL of sera-mag magnetic streptavidin-coated beads (Ref.# 30152103010150, Cytiva) at RT for 2 h and stabilized overnight at 4 °C. After washing two times with 200 µL of SELEX buffer, the protein-bead complex was incubated with the 6-FAM-labeled aptamer of the concentration 3, 10, 30, 100, 300, and 1000 nM, respectively for 2 h. The unbound aptamer was removed by washing three times with 200 µL of SELEX buffer; the bound aptamer was eluted using 30 µL of hot SELEX buffer at 95 °C. The fluorescence intensity from the sample at 520 nm was collected using the Clariostar microplate reader (BMG Labtech) and plotted against concentration using origin software to calculate the binding affinity (*K_d_*).

**Synthesis of gold nanoparticles.** A three-necked round bottom flask was cleaned with freshly prepared aqua regia (concentrated HNO_3_ and HCl in 1:3 molar ratio), rinsed with nuclease-free water, and perfectly dried before use. The AuNPs colloid was synthesized from KAuCl_4_ (Ref. # 334545-1G; Sigma Aldrich) precursor using the classical citrate reduction method 111. Briefly, a 100 mL of 1 mM KAuCl_4_ solution was heated to boiling. Then, 2 mL of 194 mM sodium citrate solution (CAS # 1545801, Sigma Aldrich) was added and boiled for an additional 15 min with good stirring. The color of the solution changes from yellow, clear/gray, and finally to dark wine-red. After 15 min of boiling the reaction, the flask was taken out and cooled slowly to room temperature.

**Flow cytometry experiment to measure the binding potential of the aptamers to WT spike-protein.** The 100 μL of 500 nM his-tagged target protein was prepared in SELEX buffer and incubated with 1 μL of Ni-NTA magnetic beads (G-biosciences, # Ref. 062N-A), rotating for 1 h at RT. The bead/protein complex was washed twice with 200 μL of SELEX buffer. Then, 50 pmol of 6-FAM-labeled aptamer was prepared in 100 μL of SELEX buffer and incubated with the complex of protein and Ni-NTA beads for 1h at RT with rotation. After incubation, the beads were washed two times with 200 μL of SELEX buffer and finally resuspended with 100 μL of SELEX buffer. The fluorescence emission produced by the 6-FAM-labeled aptamers bound on the complex of protein and Ni-NTA bead were analyzed by flow cytometer measurement (Guava easyCyte 5HT, Catalog # 0500-4005).

**Flow cytometry experiment to measure the binding affinity of the S1B6C3-A5-A2A2C1 fusion aptamer.** The 50 μL of 200 nM His-tagged spike-protein was prepared in SELEX buffer and incubated with 1 μL of Ni-NTA magnetic beads (2×diluted, G-Biosciences, Ref. # 062N-A), rotating for 1 h at RT. The bead/protein complex was then washed twice with 100 μL of SELEX buffer. Then, 1, 3, 10, 30, 100, 300, and 1000 nM solutions of 6-FAM-labeled aptamer were prepared in 50 μL of SELEX buffer and incubated with the bead/protein for 1h at RT with rotation. After incubation, the beads were washed twice with 100 μL of SELEX buffer and finally resuspended with 50 μL of SELEX buffer. The 6-FAM-labeled aptamers bound on protein/bead complex were then analyzed by flow cytometry (Guava easyCyte 5HT, Catalog # 0500-4005). Each experiment was performed for three trials, and flow events were integrated to calculate average fluorescence emission and the binding affinity (*K_d_*).

## Supplementary Material

Supplementary figures and tables.
