# Supplementary material for: DNA aptamers inhibit SARS-CoV-2 spike-protein binding to hACE2 by an RBD- independent or dependent approach
Source: Theranostics. 2022 Jul 18;12(12):5522–36. doi: 10.7150/thno.74428 (PMC9330529; doi:10.7150/thno.74428)
Supplement: Supplementary file 1 — Supplementary figures and tables. [file thnov12p5522s1.pdf]

# DNA aptamers inhibit SARS-CoV-2 spike-protein binding to hACE2 by an RBD-independent or dependent approach

Achut Prasad Silwal, Siddhartha Kalpa Samadhi Thennakoon, Satya Prakash Arya, Rick Mason Postema, Raunak Jahan, Chien Minh Tran Phuoc, and Xiaohong Tan*

Department of Chemistry and Center for Photochemical Sciences, Bowling Green State University

*To whom correspondence should be addressed: tanx@bgsu.edu

***Supporting Information***

**Supporting Figures:**

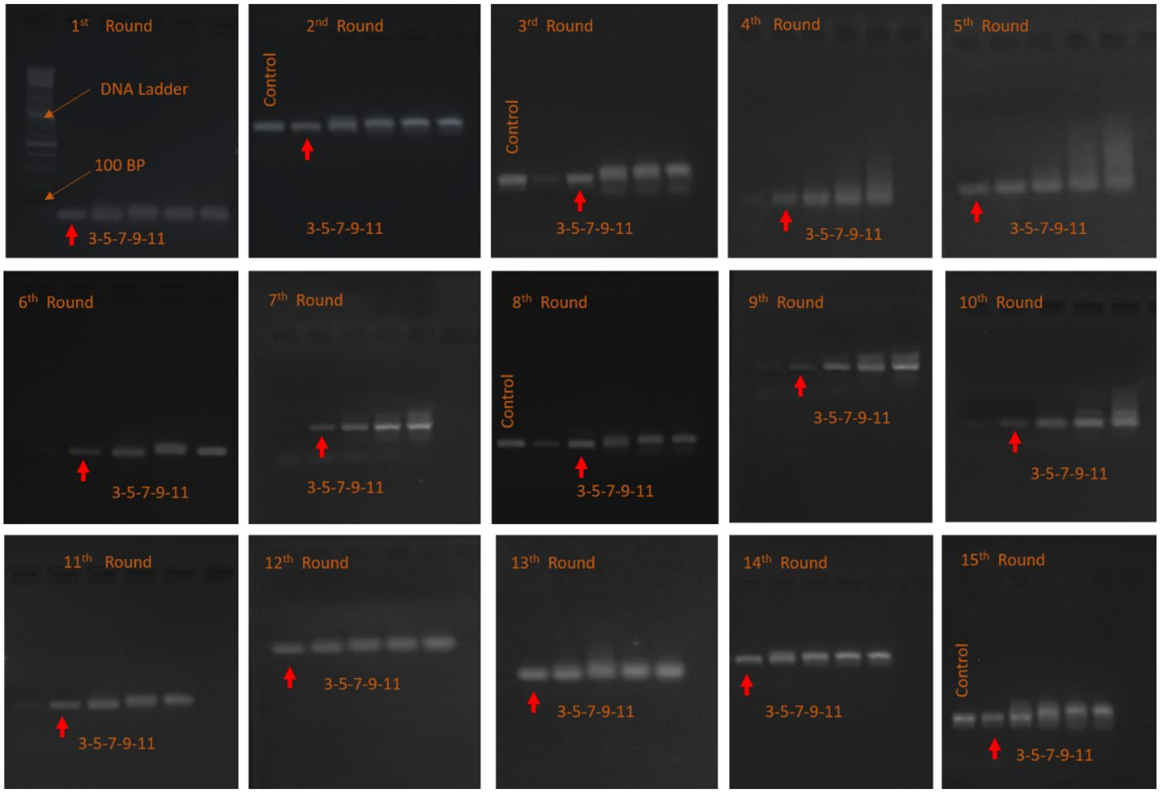


**Figure S1.** The gel electrophoresis data assesses the quality of the PCR product to determine the optimized number of PCR cycles (indicated by upward red arrow) required for the DNA amplification. The correct size of the PCR product has 73 base pairs of nucleotides. The DNA ladder or the control (correct PCR product from first selection) was used to characterize the size of the PCR products. Selection round 1-5 has additional 9 PCR cycles, and 6-15 has additional 7 PCR cycles run before the optimization.

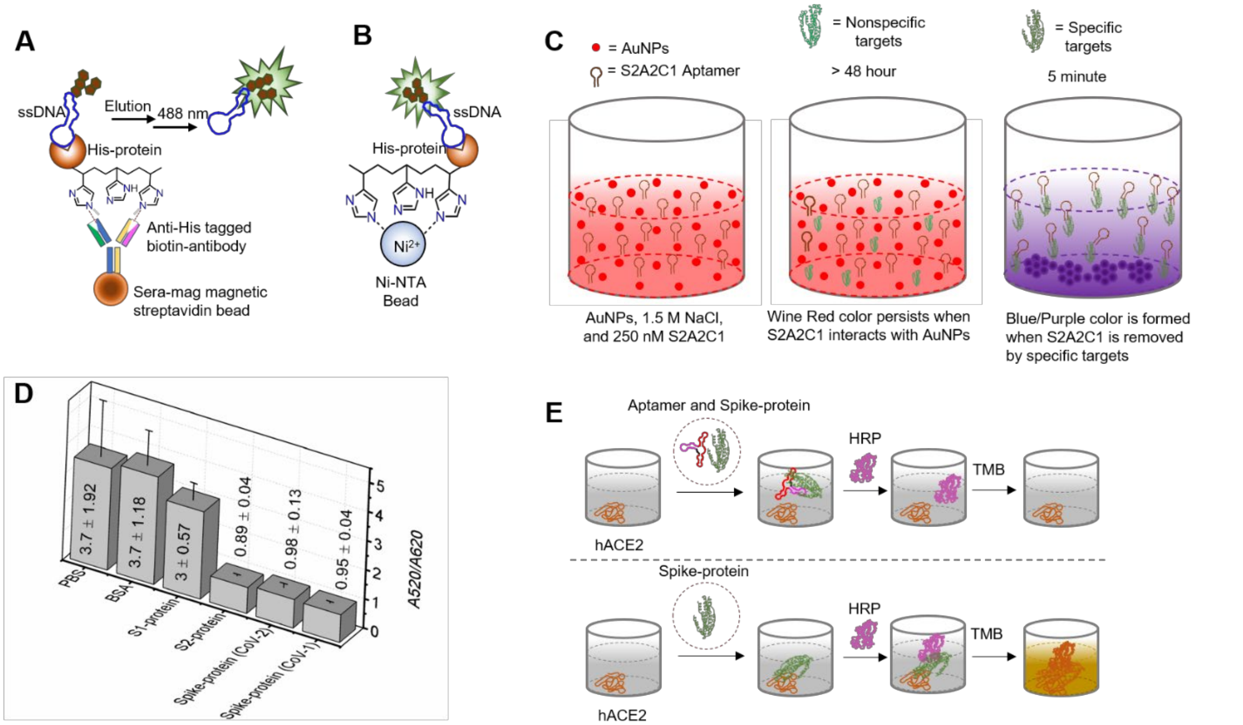


**Figure S2.** (A) Schematic representation of 6-FAM-labeled DNA binding to the target-protein/bead complex (not to scale). (B) Schematic representation of 6-FAM-labeled DNA bound to the target protein for fluorescence microscopy. (C) Conceptual scheme showing that S2A2C1 avoids aggregation and preserves the red wine color of the AuNPs colloids (left panel). When the specific targets (S2- or spike-proteins) are added, S2A2C1 preferentially binds to the specific target, leaving AuNPs for aggregation and producing the purple color (right panel). (D) The ratio of the absorbances (A520 nm/620 nm) quantifies specific binding. When A520 nm/620 nm is smaller than 1, it indicates specific binding, while a larger ratio indicates the absence or weaker binding. (E) Schematic representation of the ELISA-based inhibition efficacy measurement (not to scale). First, the well of the high binding 96 well plates were coated with 50 µL solution of 10 µg/mL hACE2 by overnight incubation at 4 °C. Then the hACE2 solution was removed and 100 µL blocking solution (5 mg/mL solution of BSA) was added and incubated for 1 h. After removing blocking solution and washing for 3 times, the premixed solution of aptamers and His-tagged spike-protein was added into the well plate. When an aptamer inhibits the His-tagged spike-protein/hACE2 interaction, the anti-His tagged HRP no longer persists after washing and does not impart color to the TMB (upper panel). When no aptamer or unspecific aptamer is present with the spike-protein, a color product was formed (lower panel). The absorbance from the chromogenic reagent (HRP+TMB) is measured by the Clariostar microplate reader. The higher absorbance value from the chromogenic reagent indicates the lower inhibition efficacy of the aptamers.

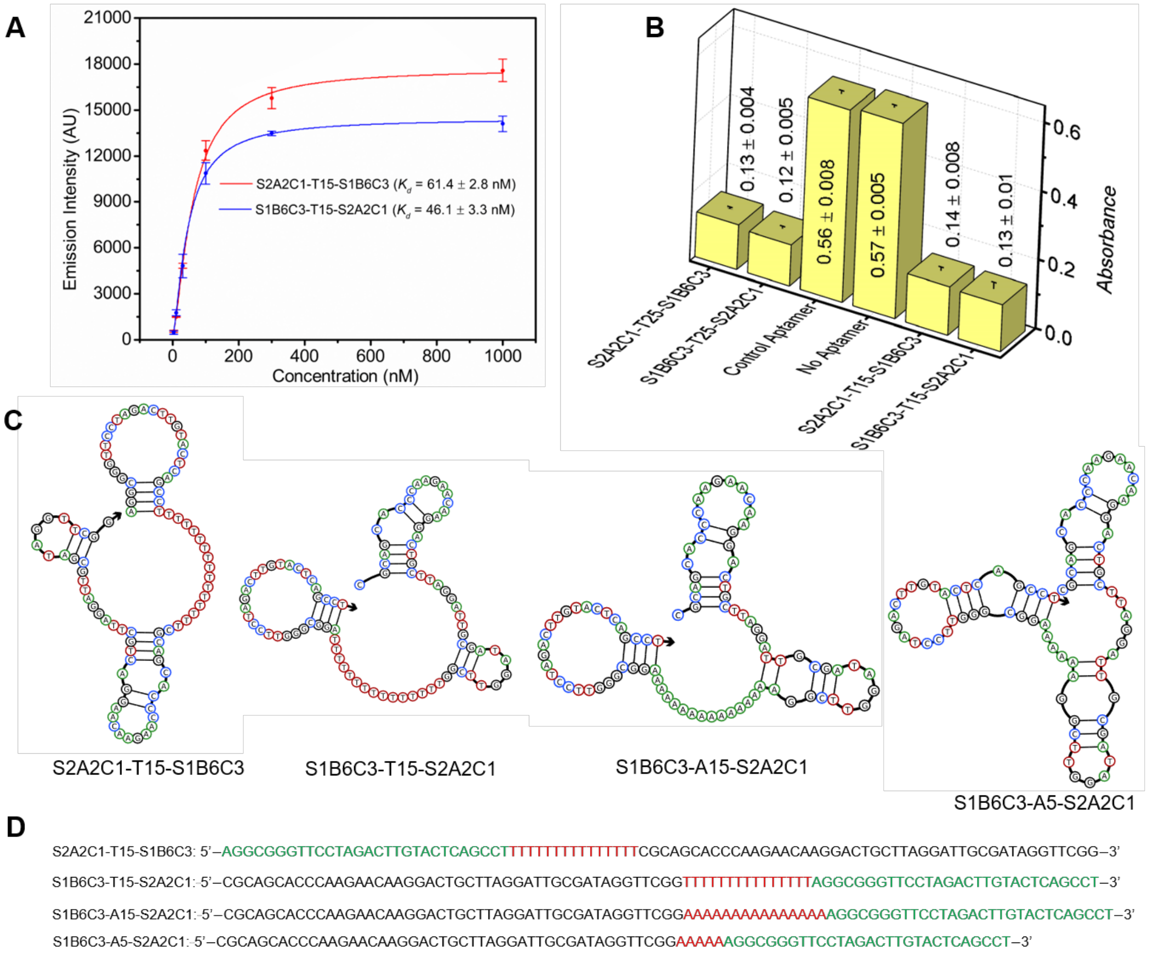


**Figure S3.** (A) The binding affinity of two fusion aptamers, S2A2C1-T15-S1B6C3 and S1B6C3-T15-S2A2C1. The fusion aptamer having S1B6C3 in the 5' position has better binding affinity than that in the 3' position. (B) The neutralization efficacy of fusion aptamers having S1B6C3 in the 5' or 3' position for polyT linker (T15 and T25) against hACE2/spike-protein interaction. Results show the S1B6C3 in the 5' position is more compelling. (C) Secondary structures of the fusion aptamers by NUPACK and (D) Nucleotide sequences.

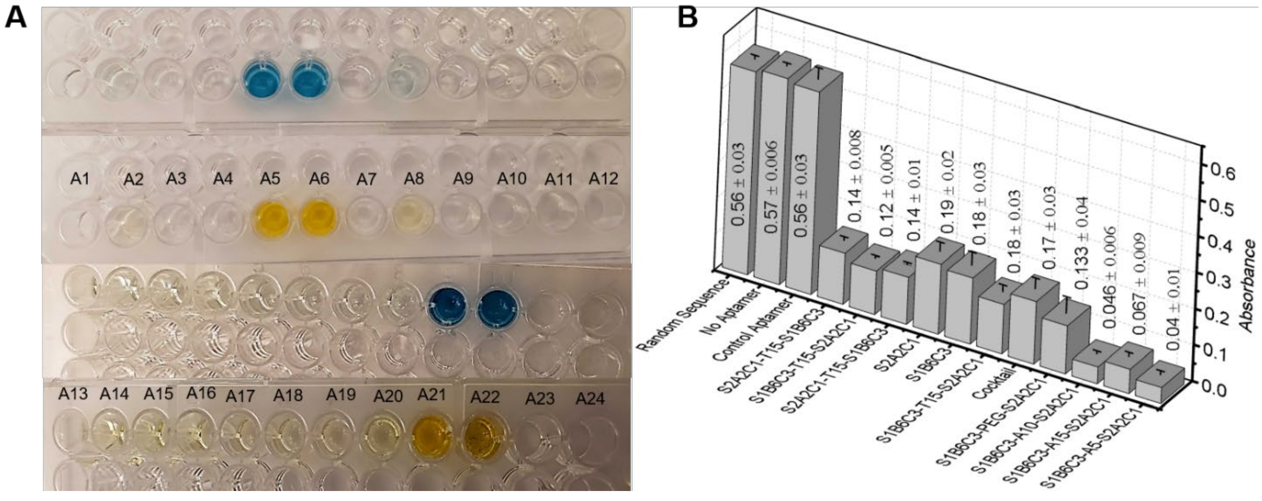


**Figure S4.** The ELISA data determines the neutralization efficacy of the aptamers against the WT spike-protein. (A) The well plate showing the intensity of the color product formed due to HRP mediated oxidation of TMB, the top and bottom panel are obtained from same sample before and after the use of 2 µL of Conc. $H_2SO_4$ respectively. The sample description is as following. A1 = Cocktail, A2 = S1B6C3-A5-S2A2C1, A3 = S2A2C1, A4 = S1B6C3-A10-S2A2C1, A5 = control aptamer, A6 = No aptamer, A7 = S2A2C1-T15-S1B6C3, A8 = S1B6C3, A9 = S1B6C3-T15-S2A2C1, A10 = S1B6C3-PEG-S2A2C1, A11 = TMB, A12 = SELEX Buffer, A13, A14=S1B6C3-T15-S2A2C1, A15, A16 = S1B6C3-T25-S2A2C1, A17, A18 = S2A2C1-T25-S1B6C3, A19, A20 = S2A2C1-T25-S1S2A1C, A21, A22 = No aptamer, A23, A24 = TMB. (B) The absorbance from the yellow color measured at $\lambda_{max}$ = 450 nm. The neutralization efficacy of the aptamers is analyzed as an inversely related property of the absorbance.

A

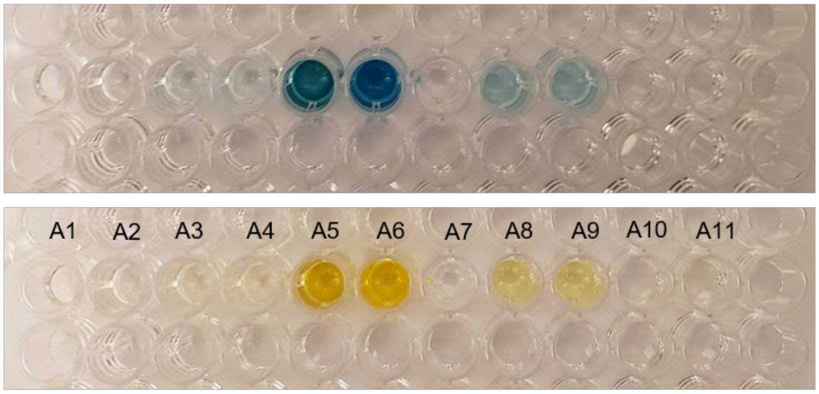


B

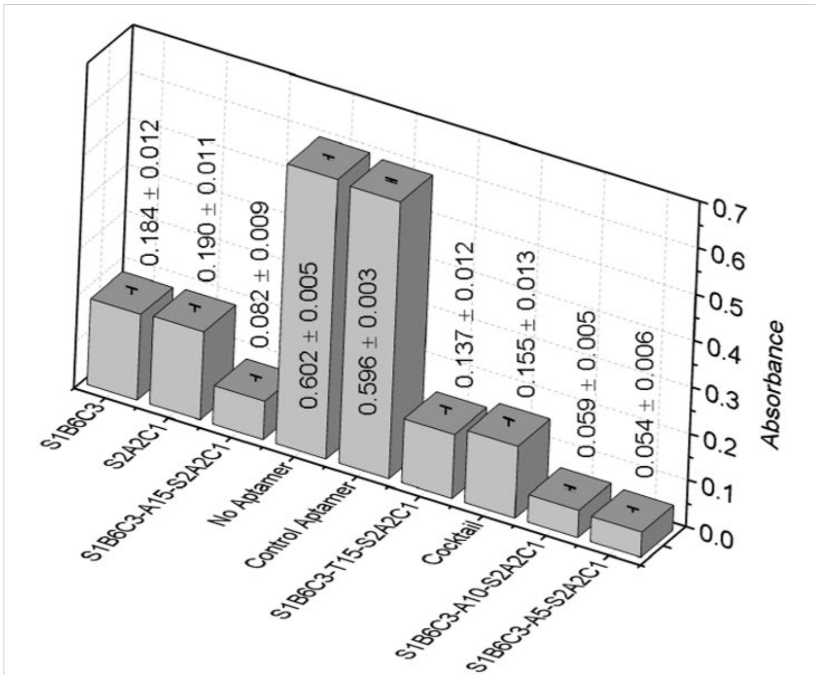


**Figure S5.** The ELISA data determines the neutralization efficacy of the aptamers against the Delta spike-protein. (A) The well plate showing the intensity of the color product formed due to HRP mediated oxidation of TMB, the top and bottom panel are obtained from same sample before and after the use of 2 μL of Conc. $H_2SO_4$ respectively. The sample description is as following. A1 = S1B6C3-A5-S2A2C1, A2 = S1B6C3-A10-S2A2C1, A3 = cocktail, A4 =S1B6C3-T15-S2A2C1, A5= control aptamer, A6 = no aptamer, A8 = S1B6C3-A15-S2A2C1, A9 = SELEX Buffer, A8 = S2A2C1, A9 = S1B6C3, A10 = TMB, A11=SELEX buffer. (B) The Absorbance from the yellow color measured at $\lambda_{max}$ = 450 nm. The neutralization efficacy of the aptamers is analyzed as an inversely related property of the absorbance.

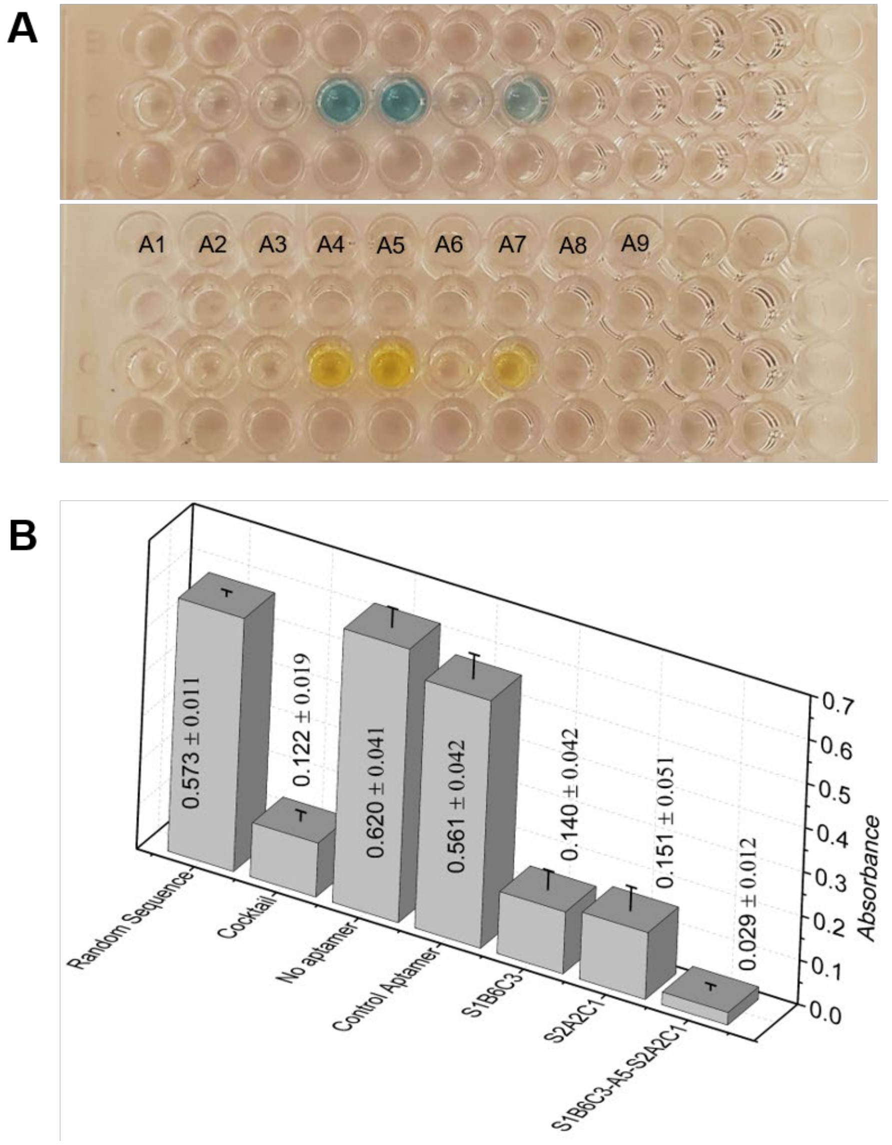


**Figure S6.** The ELISA data determines the neutralization efficacy of the aptamers against the Omicron spike-protein. (A) The well plate showing the intensity of the color product formed due to HRP mediated oxidation of TMB, the top and bottom panels were obtained from same sample before and after the use of 2 μL of Conc. H2SO4 respectively. The sample description is as following. A1 = S1B6C3-A5-S2A2C1, A2 = S2A2C1, A3 = S1B6C3, A4 = Random Sequence, A5 = no aptamer, A6 = cocktail, A7 = Control Aptamer, A8 = TMB, A9 = SELEX Buffer. (B) The Absorbance from the yellow color measured at $\lambda_{max}$ = 450 nm. The neutralization efficacy of the aptamers is analyzed as an inversely related property of the absorbance.

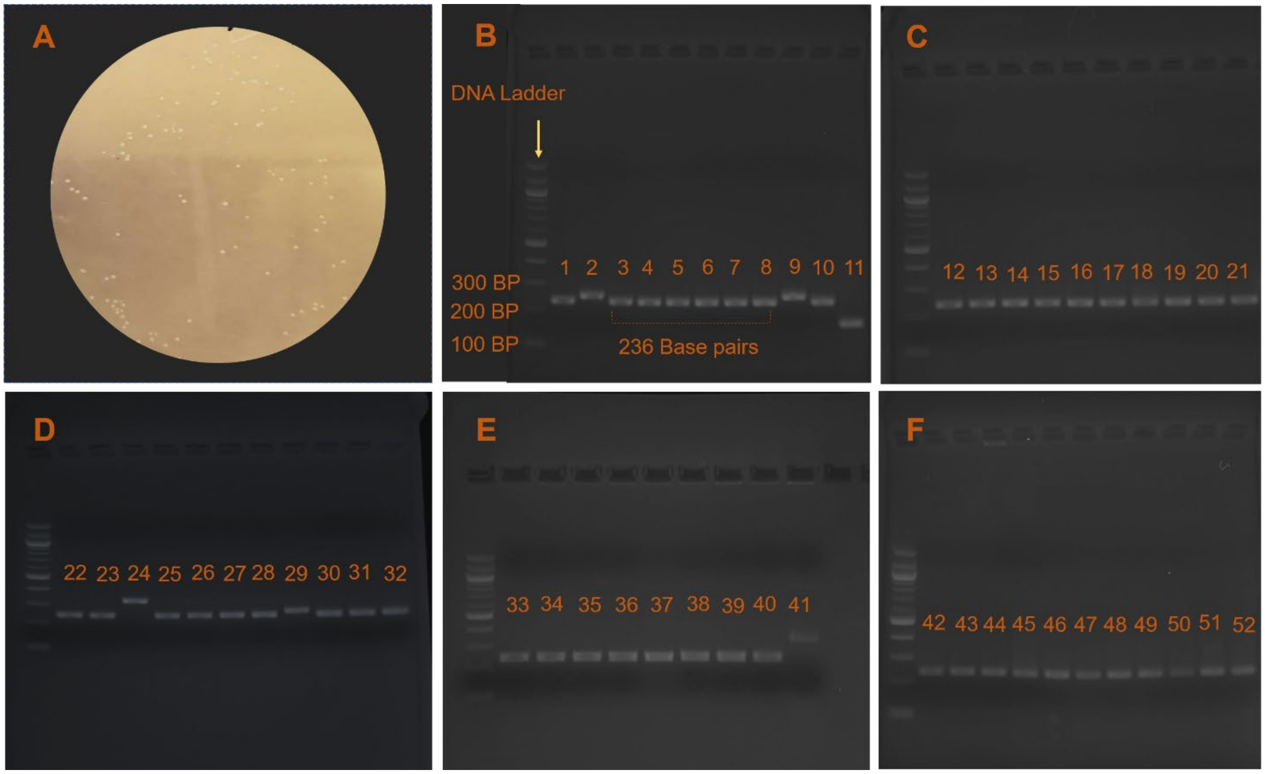


**Figure S7:** (A) The colonies of the ampicillin resistant *E. coli* bacteria; the purified dsDNA received from the 15th round of selection was ligated with TOPO vector, and the recombinant DNA was used to transform the *E. coli* component cell. The single colony of the bacterial transferred to culture the bacteria in Luria broth solution. (B-F) The gel electrophoresis data of the bacterial PCR product, the desirable insert has 236 nucleotides. The 1μL of bacterial culture was used for the PCR and remaining was used to extract the purified plasmid for the DNA sequencing. We measured the concentration of the purified plasmid and used 40 plasmid samples with higher concentration for the DNA sequencing.

**Table S1: The Sequence obtained from 40 purified plasmid samples. The randomized sequences of the aptamers are flanked by reverse and forward primer on their 5' and 3' ends respectively as represented by** 5'-CAAGGAGCGACCAGAGG-N40-TGGCATCCTTCAG CCC-3'.

| Aptamer | Sequences | Repetitions | Percentage (%) |
|---|---|---|---|
| S2A1 | 5'-CAAGGAGCGACCAGAGGGGCGGTTTATCAACAACTCG CTCTGTACACCACTCTTTGTTGGCATCCTTCAGCCC-3' | 8 | 20 |
| S2A2 | 5'-CAAGGAGCGACCAGAGGCGGGTTCCTAGACTTGTACTC AGCCTTTACAGCTATGCCCTGGCATCCTTCAGCCC-3' | 23 | 57.5 |
| S2A3 | 5'-CAAGGAGCGACCAGAGGGGCGGTTTATTAACAACTCGC TCTGTACACCACTCTTTGTTGGCATCCTTCAGCCC-3' | 2 | 5 |
| S2A4 | 5'-CAAGGAGCGACCAGAGGTAACTTCGACAGCTATTACCG CAACTACACCCGTCATCCGTGGCATCCTTCAGCCC-3' | 2 | 5 |
| S2A5 | 5'-CAAGGAGCGACCAGAGGCGGGTTCCTAGACTTTACTCA GCCTTTACAGGTATGCCCTGGCATCCTTCAGCCC-3' | 2 | 5 |
| S2A6 | 5'-CAAGGAGCGACCAGAGGCGGGTTCCTAGACTTGTACTC AGNCTTCACAGCTATGCCCTGGCATCCTTCAGCCC-3' | 1 | 2.5 |
| S2A7 | 5'-CAAGGAGCGACCAGAGGCCACCGTTTACCACGCTTTCT CATCCACACCCCCCGGCCATGGCATCCTTCAGCCC-3' | 1 | 2.5 |
| S2A8 | 5'-CAAGGAGCGACCAGAGGCGGGTTCCTAGACTTGTACAC AGCCTTTACAGCTACGCCCTGGCATCCTTCAGCCC-3' | 1 | 2.5 |

**Table S2: The details of the selection cycles.**

| Selection Round | S2-Protein (pmol) | Counter Selection (Ni-NTA bead/ incubation time) | Binding Time (min) | Washing repetition | Total PCR Cycles | Revised Yield (μg) |
|---|---|---|---|---|---|---|
| 1 | 100 | | 60 | 2 | 9+3= 12 | 3.9 |
| 2 | 100 | | 60 | 2 | 9+5=14 | 2.5 |
| 3 | 100 | 2 μL, 5 min | 60 | 3 | 9+4=13 | 1.7 |
| 4 | 50 | | 50 | 3 | 9+4=13 | 1.8 |
| 5 | 50 | 2 μL, 10 min | 50 | 4 | 9+3=12 | 1.9 |
| 6 | 50 | 2 μL, 10 min | 50 | 4 | 7+5=12 | 2.2 |
| 7 | 50 | | 50 | 5 | 7+5=12 | 3.0 |
| 8 | 33 | 2.5 μL, 15 min | 40 | 5 | 7+5=12 | 3.3 |
| 9 | 33 | 2.5 μL, 15 min | 40 | 6 | 7+5=12 | 3.4 |
| 10 | 33 | | 30 | 6 | 7+5=12 | 3.5 |
| 11 | 33 | 2.5 μL, 20 min | 30 | 7 | 7+5=12 | 8.6 |
| 12 | 33 | 3 μL, 20 min | 20 | 7 | 7+3=10 | 9.3 |
| 13 | 33 | | 20 | 8 | 7+3=10 | 9.6 |
| 14 | 33 | 4 μL, 25 min | 15 | 8 | 7+3=10 | 9.4 |
| 15 | 33 | | 15 | 9 | 7+3=10 | 9.6 |

**Table S3: The Sequence of the fusion aptamers associated to this work. The nucleotides base for anti-S2, and anti-S1 aptamers are shown in blue and black color respectively.**

| **Aptamer** | **Sequences** |
|---|---|
| S2A2C1-T15-S1B6C3 | 5'-AGGCGGGTTCCTAGACTTGTACTCAGCCT-T15-CGCAGCACCCAAGAACAAGGACTGCTTAGGATTGCGATAGGTTCGG-3' |
| S2A2C1-T25-S1B6C3 | 5'-AGGCGGGTTCCTAGACTTGTACTCAGCCT-T25-CGCAGCACCCAAGAACAAGGACTGCTTAGGATTGCGATAGGTTCGG-3' |
| S1B6C3-T15-S2A2C1 | 5'-CGCAGCACCCAAGAACAAGGACTGCTTAGGATTGCGATAGGTTCGG-T15-AGGCGGGTTCCTAGACTTGTACTCAGCCT-3' |
| S1B6C3-T25-S2A2C1 | 5'-CGCAGCACCCAAGAACAAGGACTGCTTAGGATTGCGATAGGTTCGG-T25-AGGCGGGTTCCTAGACTTGTACTCAGCCT-3' |
| S1B6C3-PEG-S2A2C1 | 5'-CGCAGCACCCAAGAACAAGGACTGCTTAGGATTGCGATAGGTTCGG-PEG-AGGCGGGTTCCTAGACTTGTACTCAGCCT-3' |
| S1B6C3-A5- S2A2C1 | 5'-CGCAGCACCCAAGAACAAGGACTGCTTAGGATTGCGATAGGTTCGG-A5-AGGCGGGTTCCTAGACTTGTACTCAGCCT-3' |
| S1B6C3-A10-S2A2C1 | 5'-CGCAGCACCCAAGAACAAGGACTGCTTAGGATTGCGATAGGTTCGG-A10-AGGCGGGTTCCTAGACTTGTACTCAGCCT-3' |
| S1B6C3-A15-S2A2C1 | 5'-CGCAGCACCCAAGAACAAGGACTGCTTAGGATTGCGATAGGTTCGG-A15-AGGCGGGTTCCTAGACTTGTACTCAGCCT-3' |

**Table S4: The comparison of the binding affinities measured by microplate reader and flow cytometry approaches of S1B6C3-A5-S2A2C1 aptamers against WT and Delta spike-protein.**

| Interactions | $K_d$ from clariostar microplate reader | $K_d$ from flow cytometer |
|---|---|---|
| S1B6C3-A5- S2A2C1 against WT spike-protein | 35.8 ± 4.2 nM | 34.6 ± 5.4 nM |
| S1B6C3-A5- S2A2C1 against Delta spike-protein | 34.1 ± 5.5 nM | 32.6 ± 5.7 nM |
